# Personalization of therapy of psychopathological complications of cardiac surgery in artificial circulation conditions

**DOI:** 10.1192/j.eurpsy.2024.1034

**Published:** 2024-08-27

**Authors:** M. Markova, D. Mankovsky, T. Abdriakhimova

**Affiliations:** ^1^Kharkiv National Medical University, Kharkiv; ^2^ SI “Heart Institute of the Ministry of Health of Ukraine”; ^3^Bogomolets National Medical University, Kyiv, Ukraine

## Abstract

**Introduction:**

The study of the clinical and phenomenological features of psychopathological complications of cardiac surgery (CS) in artificial circulation conditions (ACC), the development of modern approaches to early diagnosis and prognosis of psychopathology is an effective way to solve this problem has valid medical and social significance.

**Objectives:**

To increase the effectiveness of prevention of psychopathological disorders in cardiosurgical interventions based on personalization of their correction.

**Methods:**

The examination included the use of socio-demographic, instrumental, biochemical, clinical-psychopathological, psychometric, and statistical methods.

**Results:**

The study sample consisted of 700 patients who were treated by CS in ACC at the SI “Heart Institute of the MH of Ukraine”.

It was found out that the most common complication is postsurgeon cognitive dysfunction (PCD) (72.0% of patients), postsurgeon encephalopathy (PE) (31.0%) is less common, and cerebral infarction (CI) is the least common (12.2%).

It was revealed that the core psychopathological symptoms associated with CS are cognitive disorders (72.0% of the examined) and affective symptoms, represented by depressive (38.1%) and anxiety (33.9%) manifestations of mild and moderate expressiveness, and auxiliary constructs – dyssomnic (29.7%), asthenic (17.9%) and somatovegetative (9.0%) disorders. The highest prevalence of psychopathological symptoms was found in patients with CI, somewhat less in patients with PE, and the lowest in patients with PCD.

Signs of mild depressive disorder were found in patients who underwent CS in ACC, elevated levels of adynamic depression indicators, depression with fear and agitated depression, as well as increased levels of anxiety: the average level of anxiety, mental and somatic anxiety. The indicators of expressiveness of depression and anxiety in patients with CI turned out to be the highest, in patients with PE – lower, and in patients with PCD – the lowest.

We proposed a mathematical model for predicting the development of psychosocial maladjustment (PM) in patients who have undergone CS in ACC. It is based on a comprehensive assessment of three key vectors that can have a mutually potentiating pathogenetically related effect on the course of the formation of PM: surgical, neurological, and psychopathological. A complex of diagnostic, corrective and preventive measures for each of the risk groups has been developed.

**Conclusions:**

Verification of the proposed model on a representative sample of patients confirmed its high predictive ability and reliability in use.

**Disclosure of Interest:**

None Declared

